# Supplementary material for: Stress and resilience during pregnancy: A comparative study between pregnant and non-pregnant women in Ethiopia
Source: PLOS Glob Public Health. 2023 May 22;3(5):e0001416. doi: 10.1371/journal.pgph.0001416 (PMC10202304; doi:10.1371/journal.pgph.0001416)
Supplement: S1 File — (DOCX) [file pgph.0001416.s001.docx]

**Supplementary materials**

**Table A: Comparisons of mean PSS and BRS items score in pregnant and non-pregnant women**

**Table A1:** Comparisons of mean PSS items score in pregnant and non-pregnant women

| Comparison of mean stress item scores using t-test between pregnant and non-pregnant women | | | | | Linear regression (pregnancy regressed on stress) | | | | | |
| --- | --- | --- | --- | --- | --- | --- | --- | --- | --- | --- |
|  |  |  |  |  | **Unadjusted** | | | **Adjusted** | | |
| PSS-10 items | All women Mean ± SD | Pregnant women, n=166 | Non-pregnant women  n=154 | T-test  p-value | Β, estimate for pregnancy | [95% CI] | | β, estimate for pregnancy | [95% CI] | |
| Feeling upset | 1.59±0.84 | 1.60±0.90 | 1.58±0.76 | 0.91 | 0.01 | -0.20 | 0.17 | 0.19 | -0.02 | 0.41 |
| Unable to control things in life | 1.57±0.83 | 1.70±0.91 | 1.44±0.70 | 0.02 | 0.26 | -0.44 | -0.08 | 0.39 | 0.18 | 0.61 |
| Feeling nervous | 1.65±0.94 | 1.80±1.10 | 1.51±0.77 | 0.01 | 0.28 | -0.49 | -0.07 | 0.44 | 0.20 | 0.68 |
| Feeling not confident | 1.58±0.94 | 1.93±1.03 | 1.21±0.64 | 0.00 | 0.72 | -0.92 | -0.53 | 0.54 | 0.30 | 0.78 |
| Things not going your way | 1.81±0.94 | 2.15±0.91 | 1.44±0.82 | 0.00 | 0.71 | -0.90 | -0.52 | 0.34 | 0.11 | 0.57 |
| Not able to cope | 1.52±0.87 | 1.60±0.96 | 1.44±0.75 | 0.12 | 0.18 | -0.37 | 0.02 | 0.24 | 0.01 | 0.47 |
| Being irritable | 1.81±0.90 | 2.16±0.98 | 1.45±0.62 | 0.00 | 0.71 | -0.89 | -0.53 | 0.50 | 0.27 | 0.72 |
| Not on top of things | 1.83±0.93 | 2.21±0.98 | 1.42±0.66 | 0.00 | 0.79 | -0.98 | -0.61 | 0.61 | 0.38 | 0.84 |
| Feeling angry | 1.71±0.89 | 1.87±1.00 | 1.55±0.73 | 0.01 | 0.33 | -0.52 | -0.13 | 0.39 | 0.15 | 0.63 |
| Difficulties getting high | 1.58±0.84 | 1.73±0.97 | 1.42±0.64 | 0.01 | 0.31 | -0.49 | -0.13 | 0.35 | 0.13 | 0.57 |

**Table A2:** Comparisons of mean BRS items score in pregnant and non-pregnant women

| Comparison of mean resilience item scores using t-test between pregnant and non-pregnant women | | | | | Linear regression (pregnancy regressed on resilience) | | | | | |
| --- | --- | --- | --- | --- | --- | --- | --- | --- | --- | --- |
|  |  |  |  |  | **Unadjusted** | | | **Adjusted** | | |
| BRS-6 items | All women  Mean ± SD | Pregnant women, n-166 | Non-pregnant women n=154 | T-test  p-value | β, estimate for pregnancy | [95% CI] | | β, estimate for pregnancy | [95% CI] | |
| Bounce back quickly | 3.14±1.29 | 2.61±1.28 | 3.72±1.03 | 0.00 | -1.11 | -0.88 | -1.37 | -1.09 | -1.40 | -0.79 |
| Not hard time during time of stress | 3.44±1.13 | 3.38±1.21 | 3.51±1.05 | 0.27 | -0.12 | -0.13 | 0.37 | -0.01 | -0.30 | 0.32 |
| Nit difficult to recover from stressors | 3.05±1.21 | 2.70±1.25 | 3.43±1.05 | 0.00 | -0.73 | 0.47 | 0.98 | -0.56 | -0.87 | -0.25 |
| Not hard to get back from something bad | 3.31±1.23 | 3.10±1.32 | 3.56±1.07 | 0.00 | -0.49 | 0.22 | 0.75 | -0.40 | -0.73 | -0.06 |
| Little trouble to come through difficulties | 3.16±1.16 | 2.86±1.26 | 3.49±0.94 | 0.00 | -0.62 | 0.38 | 0.87 | -0.60 | -0.90 | -0.29 |
| Take less time to get back to life | 3.33±1.28 | 3.08±1.40 | 3.58±1.09 | 0.00 | -0.50 | -0.78 | -0.22 | -0.35 | -0.69 | -0.01 |

**Key: P-value for t-test in simple analysis,** β **– estimate for pregnancy in regression analysis**

**Table B: Association of pregnancy with stress and resilience score**

**Table B1: Association of pregnancy with stress score**

| **Regressed for stress** | | **Model 5a** | | |
| --- | --- | --- | --- | --- |
| **Exposures** | Categories | β | [95% CI] | |
| Pregnancy status | Pregnant | **2.83** | **1.74** | **3.91** |
| Age | Age | -0.06 | -0.17 | 0.05 |
| Parity | Parity>2 | -0.14 | -1.20 | 0.91 |
| Marital | Non-married | 0.61 | -1.14 | 2.36 |
| Social support | Good support | -0.04 | -1.06 | 0.97 |
| Religion | Non-Muslim | -0.03 | -0.95 | 0.89 |
| Family size | Family size >5 | 0.28 | -0.85 | 1.40 |
| Income | Low income | 0 |  |  |
|  | Medium income | -0.19 | -1.56 | 1.18 |
|  | Higher income | 0.01 | -1.16 | 1.15 |
| Educational status | No education | 0 |  |  |
|  | Primary | 0.93 | -0.75 | 2.62 |
|  | Secondary | 0.58 | -1.21 | 2.38 |
|  | College and above | 0.32 | -1.98 | 2.62 |
| Occupation | No occupation | 0 |  |  |
|  | Employed | -0.75 | -2.48 | 0.98 |
|  | Merchant | -.26 | -1.63 | 1.11 |
| Household food insecurity | Household food insecurity | 0.09 | -0.08 | 0.25 |
| Physical activity | As usual | 0 |  |  |
|  | Increased | **2.24** | **0.90** | **3.58** |
|  | Decreased | **1.25** | **0.26** | **2.23** |
| Distress score | Distress score | **0.25** | **0.13** | **0.37** |
| Substance use | Ever use | 0.14 | -0.82 | 1.10 |
| Resilience | Resilience score | **-0.38** | **-0.48** | **-0.28** |

**Table B2: Association of pregnancy with resilience score**

| **Regressed for resilience** | | **Model 5b** | | |
| --- | --- | --- | --- | --- |
| **Exposures** | Categories | β | [95% CI] | |
| Pregnancy status | Pregnant | **-1.64** | **-2.81** | **-0.47** |
| Age | Age | 0.01 | -0.10 | 0.12 |
| Parity | Parity≥3 | -0.18 | -1.28 | 0.95 |
| Marital | Non-married | 0.18 | -1.65 | 2.01 |
| Social support | Good support | -0.56 | -1.61 | 0.50 |
| Religion | Non-Muslim | -0.57 | -1.54 | 0.39 |
| Family size | Family size >5 | 0.48 | -0.70 | 1.66 |
| Income | Low income | 0 |  |  |
|  | Medium income | -0.70 | -2.13 | 0.73 |
|  | Higher income | -0.71 | -1.92 | 0.49 |
| Educational status | No education | 0 |  |  |
|  | Primary | -1.37 | -3.13 | 0.40 |
|  | Secondary | -1.24 | -3.11 | 0.63 |
|  | College and above | -2.08 | -4.47 | 0.32 |
| Occupation | No occupation | 0 |  |  |
|  | Employed | 0.96 | -0.85 | 2.77 |
|  | Merchant | 0.10 | -1.34 | 1.53 |
| Food insecurity | Household food insecurity | **-0.23** | **-0.40** | **-0.06** |
| Distress score | Distress score | 0.09 | -0.05 | 0.22 |
| Physical activity | As usual | 0 |  |  |
|  | Increased | **2.13** | **0.72** | **3.54** |
|  | Decreased | **1.21** | **0.18** | **2.24** |
| Substance use | Ever use | **1.38** | **0.39** | **2.38** |
| Stress | Stress score | **-0.42** | **-0.53** | **-0 .31** |

**Appendixes**

**Appendix A**: Perceived Stress Scale (PSS-10)

The questions in this scale ask you about your feelings and thoughts during the last month. In each case, you will be asked to indicate *how often* you felt or thought a certain way.

| S. No | Perceived Stress Scale (PSS-10): In the last 1-month | Never | Almost Never | Sometimes | Fairly Often | Very Often |
| --- | --- | --- | --- | --- | --- | --- |
| 1. | How often have you been upset because of something that happened unexpectedly? | 0 | 1 | 2 | 3 | 4 |
| 2. | How often have you felt that you were unable to control the important things in your life? | 0 | 1 | 2 | 3 | 4 |
| 3. | How often have you felt nervous and “stressed”? | 0 | 1 | 2 | 3 | 4 |
| 4. | How often have you felt confident about your ability to handle your personal problems? | 0 | 1 | 2 | 3 | 4 |
| 5. | How often have you felt that things were going your way? | 0 | 1 | 2 | 3 | 4 |
| 6. | How often have you found that you could not cope with all the things that you had to do? | 0 | 1 | 2 | 3 | 4 |
| 7. | How often have you been able to control irritations in your life? | 0 | 1 | 2 | 3 | 4 |
| 8. | How often have you felt that you were on top of things? | 0 | 1 | 2 | 3 | 4 |
| 9. | How often have you been angered because of things that were outside of your control? | 0 | 1 | 2 | 3 | 4 |
| 10. | How often have you felt difficulties were piling up so high that you could not overcome them? | 0 | 1 | 2 | 3 | 4 |
| Total score | |  | | | | |

**Appendix B:** Brief Resilience Scale (BRS-6)

| S. No | Brief Resilience Scale (BRS-6): Please indicate the extent to which you agree with each of the following statements | Strongly disagree | Disagree | Neutral | Agree | Strongly agree |
| --- | --- | --- | --- | --- | --- | --- |
| 1 | I tend to bounce back quickly after hard times. | 1 | 2 | 3 | 4 | 5 |
| 2 | I have a hard time making it through stressful events. | 1 | 2 | 3 | 4 | 5 |
| 3 | It does not take me long to recover from a stressful event. | 1 | 2 | 3 | 4 | 5 |
| 4 | It is hard for me to snap back when something bad happens. | 1 | 2 | 3 | 4 | 5 |
| 5 | I usually come through difficult times with little trouble. | 1 | 2 | 3 | 4 | 5 |
| 6 | Tend to take a long time to get over set-backs in my life. | 1 | 2 | 3 | 4 | 5 |
| Total score | |  | | | | |
