# Supplementary material for: Stress and resilience during pregnancy: A comparative study between pregnant and non-pregnant women in Ethiopia
Source: PLOS Glob Public Health. 2023 May 22;3(5):e0001416. doi: 10.1371/journal.pgph.0001416 (PMC10202304; doi:10.1371/journal.pgph.0001416)
Supplement: Attachment — Submitted filename: Response to Reviewers.docx [file pgph.0001416.s002.docx]

**Response to Feedback**

***Our general response:*** *The authors would like to acknowledge the reviewer/s for taking time to read and give us important feedback/comments on our manuscript. We have considered these and are grateful for the opportunity to edit and improve the manuscript. Please find below a point-by-point response to the issues raised. We have used track change in the main document to indicate changes.*

Point by point response

1. Please provide separate figure files in .tif or .eps format.

**Response: Now this is corrected and done accordingly.**

2. 2. We have noticed that you have uploaded Supporting Information files, but you have not included a list of legends. Please add a full list of legends for your Supporting Information files after the references list.

**Response: Corrected**

3. In the online submission form, you indicated that "Available on request". All PLOS journals now require all data underlying the findings described in their manuscript to be freely available to other researchers, either 1. In a public repository, 2. Within the manuscript itself, or 3. Uploaded as supplementary information.

**Response: This is now also corrected**

4. Reviewer #1: The paper presents a study of stress and resilience between pregnant and non-pregnant women in Ethiopia. The description of the study rationale, the interpretation of the results and the discussion are balanced. The paper is well-presented with a detailed statistical analysis and may attract wide interest to the readership. I have one minor concern that there is little explanation and discussion about the individual results of the hierarchical logistic analysis, which may decrease the merit of the complicated statistical analysis in this study.

**Response: Thank you for this feedback. We have now added a statement to emphasise the relevance of the different regression models and findings. “This finding is robust and associations between pregnancy and stress and resilience are consistent across the different statistical models”.**

5. Reviewer #2: This is a well written study to understand if pregnancy is associated with higher stress and lower resilience. It is great to see The associations between pregnancy with stress and resilience were stable across the different regression models indicating the robustness of the finding.
   a. Minor comments:
      1) Could higher stress and lower resistance also be associated with many of these families due to poorer education which can possible contribute to unstable households? If this data is available, it’d be great if it could be added to the study.

**Response: There was no clear pattern of an association between educational status and stress in the data. Primary level and higher level education were associated with poorer resilience compared to non-educated participants. This is indicated in the discussion section. However, another indicator of socio-economic status, household food insecurity, was associated with higher stress and lower resilience indicating that those who had food shortage/insecurity are at greater risk of stress with poor resilience. This is mentioned in the discussion section.**

2) Fig 2: labels are overlapping the figure legends and the plots

**Response: This is now corrected.**

6. Reviewer #3: The research work addresses very important aspect. Statistics methods used are proper. The data is collected form a specific area of the country and the data belongs to a limited period, has not spread all round the year. If data of all round the year could be available, then this will make the data more valuable. Month-wise analysis might also be useful.

**Response: We thank you for this feedback. Yes we do not have data all-round the year to account for seasonal effect and indicated this in the limitation section.**
