# Supplementary material for: Stress and resilience during pregnancy: A comparative study between pregnant and non-pregnant women in Ethiopia
Source: PLOS Glob Public Health. 2023 May 22;3(5):e0001416. doi: 10.1371/journal.pgph.0001416 (PMC10202304; doi:10.1371/journal.pgph.0001416)
Supplement: Attachment — Submitted filename: Response to Reviewers.docx [file pgph.0001416.s003.docx]

**Response to Feedback**

***Our general response:*** *The authors would like to acknowledge for your supportive follow up.*

1. We have noticed that you have uploaded Supporting Information files, but you have not included a list of legends. Please add a full list of legends for your Supporting Information files after the references list.

**Response:** Now we have removed the appendix from the main document and added this under the supplementary file and also we have added lists of legends at the end of the reference for this.
